# Supplementary material for: Deep Learning for Automated Elective Lymph Node Level Segmentation for Head and Neck Cancer Radiotherapy
Source: Cancers (Basel). 2022 Nov 9;14(22):5501. doi: 10.3390/cancers14225501 (PMC9688342; doi:10.3390/cancers14225501)
Supplement: Supplementary file 1 [file cancers-14-05501-s001.zip › cancers-1974697-supplementary.pdf]

Supplementary Materials: Deep Learning for Automated Elective Lymph Node Level Segmentation for Head and Neck Cancer Radiotherapy

Victor I. J. Strijbis, Max Dahele, Oliver J. Gurney-Champion, Gerrit J. Blom, Marije R. Vergeer, Berend J. Slotman and Wilko F. A. R. Verbakel

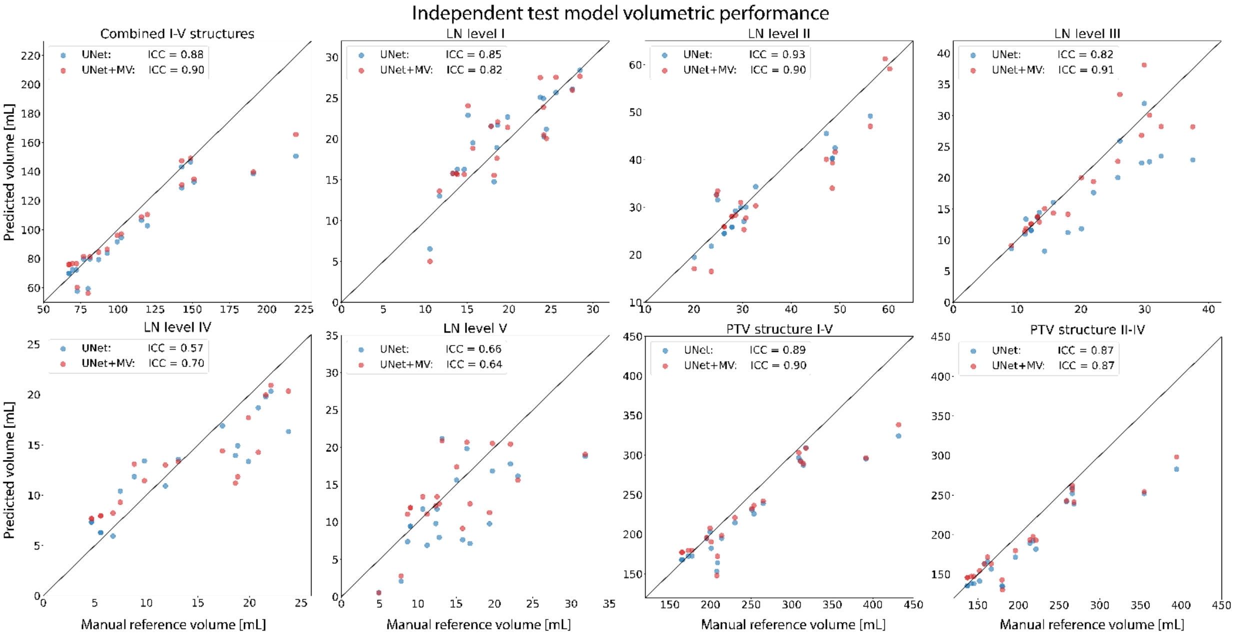

Figure S1: Predicted and manual reference volumes for all structures resulting from the independent test set. Abbreviations: ICC: intra-class correlation (two-way mixed, single measures, consistency).

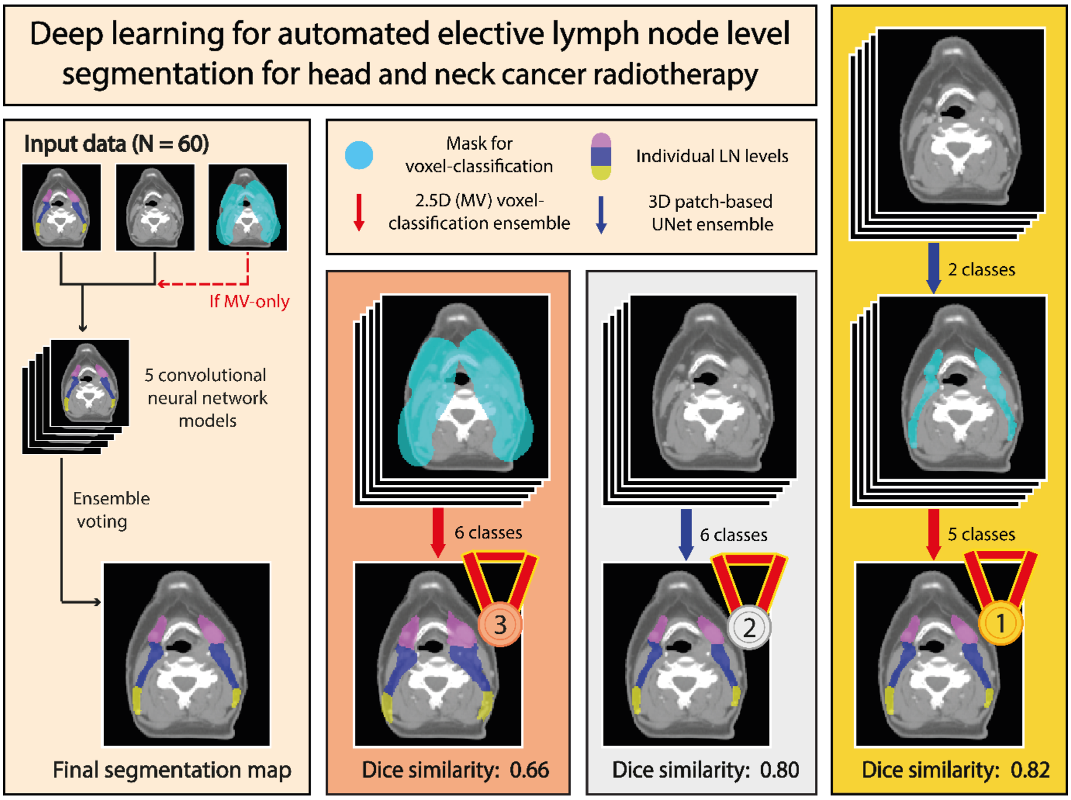

Figure S2: Graphical summary of the proposed work.
